# A Wearable Stethoscope for Long-Term Ambulatory Respiratory Health Monitoring

**DOI:** 10.3390/s20185124

**Published:** 2020-09-08

**Authors:** Gürkan Yilmaz, Michaël Rapin, Diogo Pessoa, Bruno M. Rocha, Antonio Moreira de Sousa, Roberto Rusconi, Paulo Carvalho, Josias Wacker, Rui Pedro Paiva, Olivier Chételat

**Affiliations:** 1 Swiss Center for Electronics and Microtechnology (CSEM), 2002 Neuchâtel, Switzerland; michael.rapin@csem.ch (M.R.); antonio.desousa@csem.ch (A.M.d.S.); roberto.rusconi@csem.ch (R.R.); josias.wacker@csem.ch (J.W.); olivier.chetelat@csem.ch (O.C.); 2 University of Coimbra, Centre for Informatics and Systems of the University of Coimbra, Department of Informatics Engineering, 3030-290 Coimbra, Portugal; dpessoa@dei.uc.pt (D.P.); bmrocha@dei.uc.pt (B.M.R.); carvalho@dei.uc.pt (P.C.); ruipedro@dei.uc.pt (R.P.P.)

**Keywords:** wearables, auscultation, electronic stethoscope, respiratory sound, COPD, digital health

## Abstract

Lung sounds acquired by stethoscopes are extensively used in diagnosing and differentiating respiratory diseases. Although an extensive know-how has been built to interpret these sounds and identify diseases associated with certain patterns, its effective use is limited to individual experience of practitioners. This user-dependency manifests itself as a factor impeding the digital transformation of this valuable diagnostic tool, which can improve patient outcomes by continuous long-term respiratory monitoring under real-life conditions. Particularly patients suffering from respiratory diseases with progressive nature, such as chronic obstructive pulmonary diseases, are expected to benefit from long-term monitoring. Recently, the COVID-19 pandemic has also shown the lack of respiratory monitoring systems which are ready to deploy in operational conditions while requiring minimal patient education. To address particularly the latter subject, in this article, we present a sound acquisition module which can be integrated into a dedicated garment; thus, minimizing the role of the patient for positioning the stethoscope and applying the appropriate pressure. We have implemented a diaphragm-less acousto-electric transducer by stacking a silicone rubber and a piezoelectric film to capture thoracic sounds with minimum attenuation. Furthermore, we benchmarked our device with an electronic stethoscope widely used in clinical practice to quantify its performance.

## 1. Introduction

Lung-related diseases such as pneumonia, chronic obstructive pulmonary disease (COPD), and asthma impose heavy societal and economic burdens. To date, the health state of patients with respiratory diseases is monitored intermittently only, during visits to a doctor. Therefore, it is difficult to align the medical treatment with the individual development of the disease. More frequent or even continuous observation of respiratory functions during patients’ daily life has the potential to improve the therapeutic outcome. Recent years have seen the development of a number of devices for remote recording of respiration-linked body signals. In the context of respiratory diseases, signals of interest are, among others, blood oxygen saturation, respiration frequency, body activity, and importantly, sounds produced by the respiratory organs. Sound-based remote monitoring can, for example, be done by training patients in the use of digital stethoscopes which transmit the recorded sounds to a health care professional for further analysis [1]. This approach requires an advanced level of patient compliance, as regards position accuracy and measuring frequency. Microphones attached to clothing goods are easier to use. Following this idea, free-air and lapel microphones [2] have been used to detect coughing. However, these microphones not only record the wearer’s chest sounds, but also environmental sounds, which need to be filtered out in post-processing and may pose privacy issues. Therefore, contact microphones have been integrated in patches [3] and clothing items [4] to monitor breathing and coughing. However, these attempts had limited applications such as cough frequency monitoring or respiration rate monitoring, as either the sensor quality (i.e., sensitivity, bandwidth) or the integration quality could not match the conventional stethoscope auscultation to diagnose respiratory diseases. In this study, we present a novel contact microphone assembly, with a very thin form factor, show its design and experimentally validate its characteristics to address these two challenges.

The wearable stethoscope presented in this paper was designed in the frame of the European project WELMO, which aims at developing and validating a new generation of low-cost and low-power miniaturized sensors. The sensors are integrated in a comfortable vest, enabling the effective and accurate monitoring of the lungs. The goal of the WELMO vest is to measure activity, bioimpedance and chest sounds with a high spatial resolution. The key to improve the wearing comfort of systems with many sensors, is to reduce the cabling between sensors. In WELMO, this challenge was tackled by employing cooperative sensor technology [5], which allows incorporating in a single garment a high number of sensors with minimum cabling effort. In short, the proposed system contains up to 18 measuring sensors and 1 master unit. All sensors are connected via a two-wire bus only (non-shielded wires). The data acquired in the measuring sensors is first concentrated in the master unit and then wirelessly transferred to an external unit. This results, without any impact on the signal quality, in systems with easier integration, improved comfort and higher reliability. Finally, bioimpedance is measured with dry electrodes to maximize the system’s ease-of-use and wearing comfort.

In this article, we focus on the design and characterization of the sensor module which acquires thoracic sounds. The central element of the sensor module, the acoustic transducer, can either be realized by using an air microphone [6,7,8] or a contact microphone [9,10,11]. Air microphones (electret condenser microphones and capacitive or piezoelectric MEMS microphones) are commonly inserted into the stethoscope heads [8] in order to benefit from an optimized mechanical design to guide the pressure waves. The work [12] provides a detailed analysis on the impact of different mechanical designs. While the utilization of air microphones necessitates a diaphragm, contact microphones can directly be interfaced with the skin. In the context of long-term multi-channel lung sound monitoring, positioning of the sensors and their stabilization are also studied—which is not a primary concern for electronic stethoscopes as they are intended to be used by a clinician. Positioning of the transducers on the posterior chest defines which pulmonary diseases are of interest for diagnosis. The works [7,8] employ slightly different patterns for placing the transducers, yet all these systems incorporate more transducers close to the diaphragm than close to the neck as a natural result of the respiratory system geometry. Stabilization of the sensors is critical as it reduces the low-frequency noise stemming from movement artefacts. In order to stabilize the transducers on the skin, using adhesive tapes has been preferred due to its simplicity [13]. However, placing the transducers into a foam pad [8], [14] is shown to benefit from both better stabilization and, with proper material selection, ambient noise reduction. The work [8] reports attenuation superiority of a foam pad compared to adhesive tape approach, which can reach up to 50 dB difference.

## 2. Thoracic Sound Acquisition Module Design

This section reports the design of the thoracic sound acquisition module. The module serves to capture the pressure waves, originating from the (turbulent) airflow in the respiratory tract, and to convert these to electrical signals which can then be communicated to the central unit as digital data. The signal flow path begins with the coupling of pressure waves to the module’s physical interface with the body, and continues with the conversion of vibration signals to electrical signals via a piezoelectric transducer, conditioning of the analog signals, and finally ends with on-site quantization of these signals. Accordingly, the sound acquisition module consists of a piezoelectric transducer and read-out electronics. Coherent with the signal flow path, first the transducer design is presented and in the second part of the section, the design of the electronics is detailed.

### 2.1. Transducer Design

Sound waves emanating from airflow in the respiratory organs propagate omnidirectionally, though not necessarily with the same frequency response, towards the outer surface of the body. These waves, although being within the frequency range humans can perceive, cannot be captured like speech or any other airborne sound. This is essentially due to the incompatibility of the two media, body and air, or more precisely due to the acoustic impedance mismatch between these two media. Impedance matching is a well-known concept that is applied in different physical domains (electronics, optics, acoustics, etc.) to maximize the energy transfer from one point to another and particularly from a source to a load. In the case of an impedance mismatch, a fraction of the incoming wave is reflected back towards the source; thus, limiting the signal power on the receiving end. For instance, in conventional acoustic stethoscopes, this problem manifests itself at each junction, i.e., at the chest wall and the stethoscope’s diaphragm, at the tube and the earpiece, etc. [15]. Bell (or horn) shaped stethoscope body parts connecting the large diaphragm to the narrow meatus in the early stethoscopes can be given as an example of an attempt of impedance matching [16].

Recent work reported a silicone membrane filled with water (or hydrogel) to acquire phonocardiogram signals [10]. Although the widespread use of the proposed method is arguable, due to fabrication complexity and encapsulated liquid, it highlights the significance of impedance matching between propagation media. Considering that the acoustic impedance of water is quite close to that of the body (taking an average of different tissues), we may approximate this silicone mold encapsulating water as an extension of the body. Such an extension can be described as a tapered impedance matching from the source to the load, i.e., to the air microphone, analogous to bell-shaped (or conical) stethoscope heads. Klum et al. [6] propose a method to improve the sensitivity of the system through amplification by coupling a commercial stethoscope diaphragm to an air microphone. However, a simple calculation based on the ratio of the diaphragm surface and the microphone aperture provides a very optimistic result in terms of amplification as some essential basic assumptions of such calculation, namely rigid walls, freely moving piston with zero mass, are practically not fulfilled [17].

In this work, we present a method which does not involve air as a propagation medium since the introduction of electronic stethoscopes has eliminated the necessity to guide the thoracic sounds all the way through from the chest wall to the ear. Today’s electronic stethoscopes work on the principle of on-site signal acquisition and digitization—thus improving the noise immunity in the subsequent stages. Although conventional tubes and earpieces are generally preserved in most of the electronic stethoscopes in the market, mainly to be compliant with the habits of clinicians, the sound is actually regenerated by means of a speaker driven by the input signals acquired at the stethoscope head piece. On top of that, for applications involving acquisition of thoracic sounds from multiple points at the same time, preserving the hearing experience becomes less important—or a feature than can be considered as nice-to-have.

An important disadvantage of an air layer between the chest wall and the sensing unit is the high sensitivity of such systems to ambient noise. Unless a very rigid wall or multi-layer sound absorption solutions are incorporated, the ambient noise will penetrate through the sidewalls into the “air chamber” and decrease the signal quality. Considering the wavelength of the sound signals at the frequencies of interest (100–2000 Hz), the absorption layer needs to be impractically thick for the presented application.

Based on the arguments listed above, in this work, we have implemented a diaphragm-less acousto-electric transducer by stacking a silicone rubber and a piezoelectric film—which is also known as contact microphone. The silicone rubber plays two roles: (1) providing the physical interface with the skin and (2) supporting and shaping the piezoelectric film. In conventional acoustic stethoscopes, a diaphragm is employed to interface with the skin. When the diaphragm, conforming to the skin, moves up and down, the pressure inside the cavity of the stethoscope changes and this pressure difference is transmitted to the listener’s ear [17]. In the proposed implementation, sound waves travel through the silicone rubber till the piezoelectric film, which itself may be approximated as a diaphragm due to its particular mechanical fixation. The silicone rubber with a similar specific acoustic impedance as the body’s not only provides a good acoustic impedance matching, which allows considering the silicone part as a continuation of the body [18], but also provides an inherent barrier against the ambient noise impinging from side walls as its specific acoustic impedance is significantly different than that of the air. From the perspective of sensor design, employing such a silicone rubber brings another advantage: we can consider the body and the silicone rubber as the same objects; thus, providing a semi-infinite medium [18] which consequently makes the analytical calculation of radiation resistance; thus, the damping factor, more practical [19].

A piezoelectric thin film, conforming to the surface of the silicone rubber, converts the pressure wave impinging on the material to an electrical signal whose amplitude is proportional to the strain. Polyvinylidene difluoride (PVDF or PVF2) is employed to realize the sensing unit. Since the demonstration of the piezoelectric characteristics of PVDF, the material has been widely used in acoustic applications, particularly for underwater acoustics thanks to its specific acoustic impedance quite close to that of the water. Thanks to this property, no impedance matching layer between the two media is necessary. Considering that the acoustic impedance of the body is quite close to that of water (mainly due to high water content), PVDF makes a perfect candidate for the presented application.

PVDF can be manufactured in the form of a thin flexible film. This quality differentiates PVDF from the widely used piezoelectric ceramics. Although the piezoelectric coefficients of PVDF are lower than those of ceramic parts, its ability to be bent or curved compensates for this disadvantage.

Comparing the longitudinal and transversal direct piezoelectric voltage coefficients (g_31_ and g_33_, respectively) of a mono-oriented PVDF hints that for a higher sensitivity, it makes more sense to use it in thickness (or transverse) mode, i.e., backing one side of the planar surface with a rigid surface and applying the pressure on the other side to modulate the thickness of the PVDF film. However, Naono et al. [20] have shown that a much higher sensitivity can be achieved in longitudinal (g_31_) mode than in thickness mode (g_33_) if one curves a PVDF film along its poling direction (1-axis) and clamps the non-curved sides (which are parallel to 2-axis) to a rigid frame. This approach provides a mechanical amplification as a function of the radius of curvature (*R*). More precisely, the sensitivity is proportional to *R*. This property comes along with a trade-off; as the sensitivity increases, the resonance frequency of the sensor decreases—thus limiting its linear operation region, i.e., the resonance frequency is inversely proportional to *R*. A simplistic model to calculate the resonance frequency is given by [20] as

(1) $$f_{0}=\;\frac{1}{2\pi R}\;\sqrt{\frac{Y}{\rho }}$$

where *Y* is Young’s modulus and *ρ* is the density of the PVDF film. It should be noted that this equation neglects the backing material and effects of clamps. Nevertheless, it is useful to understand how the resonance frequency could be controlled by tuning the design. As it is desired to acquire signals in a given bandwidth with flat frequency response, it is preferable to set the resonance frequency high enough to ensure the specified flatness in the bandwidth of interest. The flatness in audio applications is generally defined as ±2 dB (or ±3 dB) as the human ear can hardly detect a 2-dB difference.

Later Toda et al. [18] have introduced a more detailed model taking the damping due to radiation resistance and the mass loading (in this case, due to the silicone rubber) into account. Based on these calculations, in the present work, we realized the transducer with a 110 µm-thick PVDF film (TE connectivity, 3-1004346-0) with a 50 mm radius of curvature. This configuration provides a resonance frequency of 4 kHz according to the model developed in [20] and approximately 1 kHz according to the model developed in [18]. The active sensing site has an area of 120 mm^2^ (10 mm × 12 mm) which approximately corresponds to 120 pF sensor capacitance.

### 2.2. Sensor Electronics Design

This section reports the design of the electronics of the sound acquisition module. The section is organized in two subsections, first the signal conditioning for high-impedance sensors is detailed and then filtering and quantization aspects are explained.

#### 2.2.1. High-Impedance Sensor Interface

**Figure 1:**
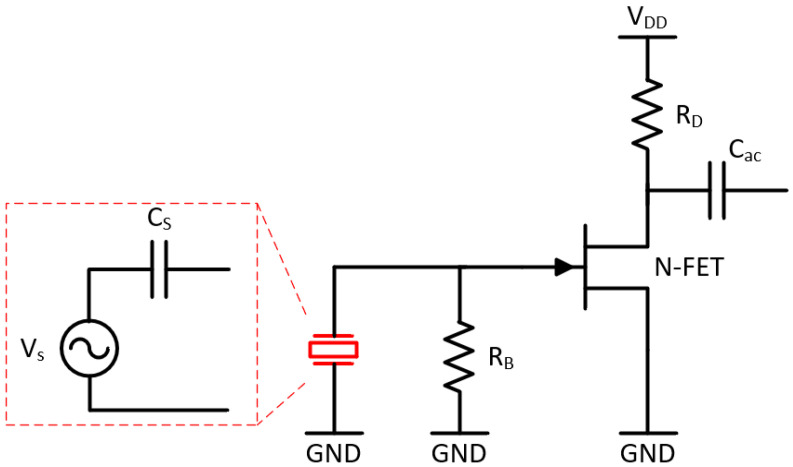
Electronic circuit (impedance converter) interfacing with the high-impedance sound sensor. The piezoelectric sensor (in red) is modelled as a voltage source in series with a capacitance in its linear mode (highlighted with red-dashed lines).

The chest sounds are picked up by the piezoelectric transducer whose behavior can be modeled as a voltage source V_S_ (with a linear voltage-strain behavior) in series with a capacitance C_s_ (see Figure 1). This configuration has the following specificities which must be taken into consideration when designing the sensor electronics: (1) Connecting the piezoelectric sensor to a resistive load results in a high-pass filter network. (2) A capacitive load (e.g., the input capacitance of an impedance converter) acts as a voltage divider. Observations (1) and (2) impose a design with a very high input impedance so that the signal amplitude and signals with low frequency are not lost [21]. (3) C_s_ acts as a high source impedance at the low frequencies the device targets. Therefore, the electronics design must consider undesired effects which typically appear with high source impedances, notably due to bias current and current noise at the input. Especially at low and moderate impedance values, the current noise is neglectable vis-à-vis the significantly more important voltage noise.

To deal with the high source impedance, we have opted for a common-source junction field effect transistor (JFET) amplifier acting as an impedance converter. JFETs are characterized by high input impedance, very low gate bias current, and low flicker (1/f) noise. Furthermore, the negative threshold voltage allows simplifying the biasing scheme by connecting the gate to the source (or to ground (GND) in the case of a common-source amplifier). As shown in Figure 1, we have realized the impedance converter (which converts the high source impedance to a lower impedance) with only one transistor to have more flexibility in the topology and component selection of the amplifier stage. The gain of the impedance converter is the product of the forward transconductance of the JFET (2SK3666-3, ON Semiconductor) and the drain resistance (R_D_). Cs and the input resistance of the impedance converter (approximately equal to the gate bias resistor R_B_) form a first order high-pass-filter (HPF).

**Figure 2:**
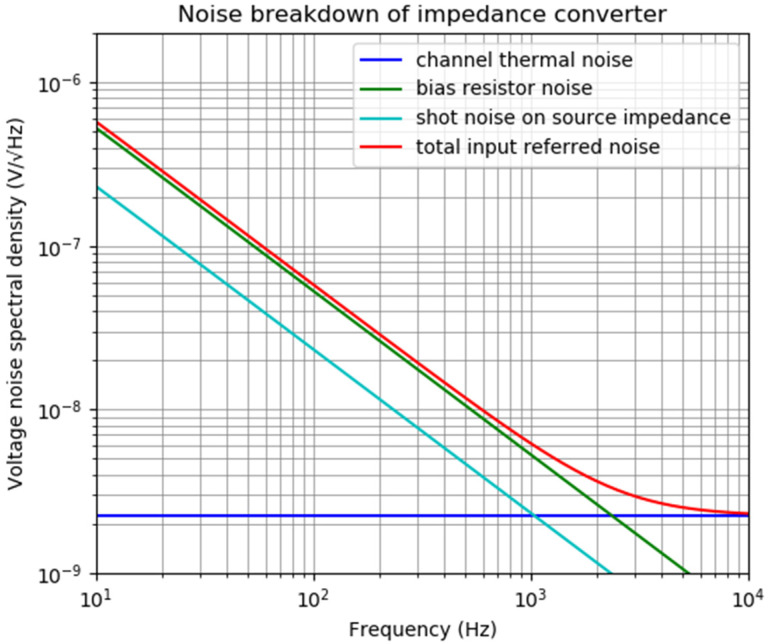
Noise breakdown of the impedance converter.

A noise analysis of this first stage shows the importance of the current noise associated with the transistor and its biasing resistor as well as the channel thermal noise. Figure 2 presents the noise breakdown of the impedance converter for a 1 GΩ bias resistance, a 10 pA input bias current, and approximately unity gain (note that at the input, a gate-to-drain capacitance C_gd_ will be seen as multiplied by (1 + gain) due to the Miller effect, which reduces the signal gain from the sensor to the drain of the JFET). For higher bias resistance values (which results in less noise), the input bias current value becomes more important and decisive for the total output noise (or input-referred noise) as well as the offset voltages. Taking into consideration that the noise voltage increases with the resistance value, it may seem at first glance that the bias resistance R_B_ should be reduced to such a value, and that the lower cut-off frequency of the amplifier (resulting from the time constant defined by the bias resistance R_B_ and capacitance of the piezoelectric sensor C_S_) is equal or slightly below the lowest frequency of interest. However, it should be noted that the network constituted by the bias resistance and capacitance of the piezoelectric sensor acts as a high-pass filter with respect to the signal to be measured, while it acts as a low-pass filter with respect to the noise generated by the bias resistance. While the time constant of the mentioned low-pass filter is proportional to the resistance, the noise voltage increases with the square root of the resistance value. As a result, noise properties of the amplifier should improve with the input resistance value. Therefore, it is preferable to have a bias resistance as large as possible to minimize the noise. Similarly, [22] formulates the input referred noise for a piezoelectric microphone interfaced with a field effect transistor (FET) and concludes that the input resistance should be as high as possible while the parasitic capacitances should be much lower than the piezoelectric sensor capacitance. It is also worth mentioning that this calculation does not take the excess noise into account which is manifested for thick film resistors when there is a DC current (in this case, bias current) passing through them.

#### 2.2.2. Signal Conditioning and Quantization

The output of the impedance converter is connected to a gain- and filtering-stage realized by an operational amplifier. In the previous section, the trade-off between transducer gain (sensitivity) and resonance frequency has been explained. As we are interested in capturing lung sounds that cover a frequency span up to 2 kHz, the signal level at the output of the impedance converter cannot reach sufficient amplitude to exploit the entire input range of the analog-to-digital converter (ADC). Therefore, a gain stage is needed between the impedance converter and the ADC. This gain stage also reduces the input-referred noise of the ADC. The answer to the imminent question why more voltage gain (A_v_) is not introduced at the impedance converter stage is the capacitive nature of the source impedance. The gate-to-drain capacitance (C_gd_) of the JFET is multiplied with a factor of (A_v_ + 1) where A_v_ is the small-signal gain of the impedance converter. The resulting capacitance is seen as a shunt capacitance from gate to ground. In the model of a piezoelectric sensor in its linear range this additional input capacitance (along with C_gs_) creates a capacitive voltage divider. Therefore, the effective gain at the output of the impedance converter is scaled with a factor of

(2) $$A_{eff}=\;\frac{C_{S}}{C_{S}+C_{gs}+\left(A_{v}+1\right)C_{gd}}$$

To avoid this attenuation, a gain is applied in the following stage. Moreover, as the designed module is intended to acquire lung sounds, filtering is applied to ensure that cardiac sounds are suppressed. Filtering has also the immediate advantage of attenuating near-DC frequencies, as it removes a significant noise contribution due to the flicker (1/f) noise of the electronics. Again, this filtering operation could also be realized at the impedance converter stage, by reducing the bias resistance R_B_; however, this approach would increase the current noise of the resistor; thus, would cause a higher voltage noise, due to integration on the source capacitance. The bandwidth of the acquisition chain is defined by a first-order high pass filter having a 3-dB cut-off frequency at ~100 Hz and a second-order low pass filter with a 3-dB cut-off frequency at ~1.6 kHz. The flat-band gain of this stage is set to 10. The common-mode potential of the operational amplifier is set to half the supply voltage, V_DD_/2, to ensure the widest voltage swing at the output and to avoid operation close to the supply rails.

Amplified and filtered signals are quantized with a successive-approximation-register (SAR) analog-to-digital converter (ADC) providing 16-bit resolution and at a sampling rate of 5 kSamples/second (AD7988-1 from Analog Devices Inc., USA). The digital output stream is sent to a central unit via an SPI communication protocol. The central unit collects the data from all the six modules present in the wearable sensor system and transmits them to an external post-processing unit for offline signal processing.

**Figure 3:**
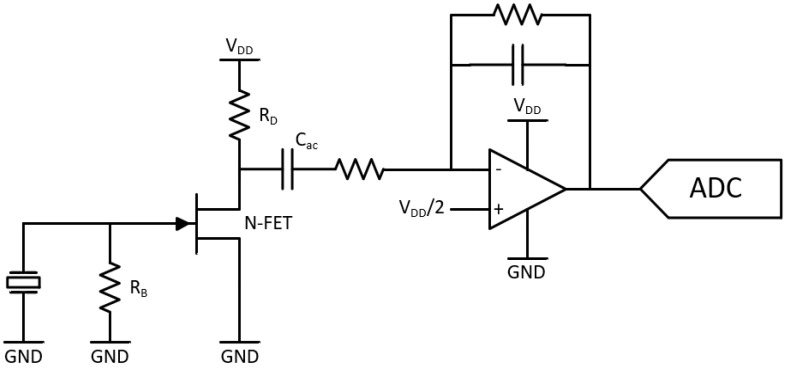
Simplified schematic of the sound acquisition module composed of impedance converter, gain and filtering stage, and analog-to-digital converter.

A simplified schematic of the sound acquisition module comprising (i) the impedance converter, (ii) gain and filtering stage, and (iii) analog-to-digital converter is shown in Figure 3.

### 2.3. Fabrication and Integration

**Figure 4:**
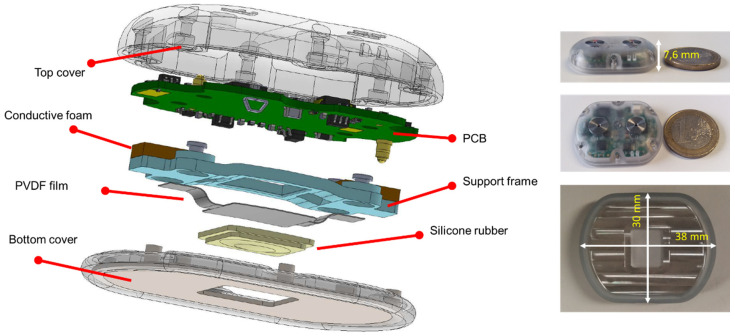
(**left**) Exploded view of the sound acquisition module and (**right**) images of the assembled sensor with its dimension; from top to bottom: side view of the sensor with 1 euro coin, top view of the sensor with 1 euro coin, and bottom view of the sensor.

Figure 4 exhibits (on the left side) the exploded view of the sound acquisition module developed based on the design principles reported in the two previous sections. The silicone rubber passing through the hole created in the bottom cover enables a direct contact with the skin. The metallic part of the bottom cover is connected to a known potential; thus, providing a shielding against electromagnetic interference, particularly 50 Hz line frequency. PVDF film has been formed using a water-jet cutter and its curvature is guaranteed by two means: the curvature of the silicone rubber and the exact replica of this curvature in the support frame. This way, the edges of the PVDF film are clamped and the required curvature is obtained. On the right hand side of Figure 4, three images of the assembled sensor is presented. The sensor has a dimension of 37 mm x 30 mm x 7.6 mm and it weighs 9.3 g.

PVDF film has a Curie temperature of about 125 °C [23]; thus, soldering or any oven treatment in the vicinity of this temperature causes “depoling”, i.e., the material loses its piezoelectric characteristics. Therefore, electrical contacts should either be created by low-temperature curing conductive epoxy or by mechanical means such as clamps or springs (e.g., a pogo pin). In this implementation, the electrical contacts between a silver-ink metallized PVDF film (TE connectivity, 3-1004346-0) and the printed circuit board (PCB) pads are realized with conductive foams (Holland Shielding). Thanks to the asymmetric pad placement, the assembly is fail-proof in terms of electrical connections and the metallization of the PVDF in contact with the silicone rubber is always connected to the ground. Grounding is critical to ensure a complete shielding of the system. The PCB is fixed to the bottom cover via plastic screws and inserts; thus, applying a force on the frame to ensure that it cannot move freely. This way, clamping of the PVDF film is also guaranteed. Finally, a plastic top cover is fixed on the PCB to complete the device. It is worth noting that the multi-layer PCB has a ground plane; thus, sandwiching the electronics and the piezoelectric sensor to complete the shielding of the system.

## 3. Evaluation and Comparative Analysis

**Figure 5:**
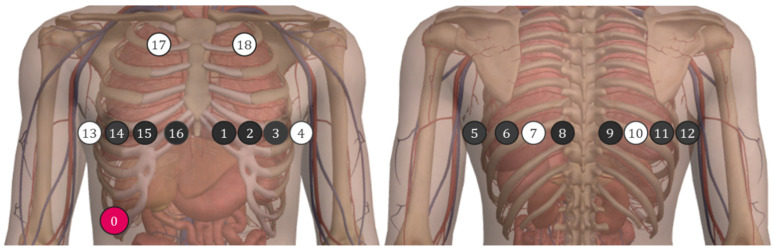
Sensor positions on the chest.

In order to characterize the sound acquired from the sensor, two typical auscultation points were considered to record respiratory sounds (positions 7 and 18 in Figure 5). Position 7 is commonly used to auscultate respiratory sounds; position 18 can be used to perform cardiac auscultation. Thus, position 18 will allow to check whether cardiac sound is a source of interference.

The respiratory sounds were simultaneously recorded with a 3M™ Littmann^®^ Electronic Stethoscope Model 3200 (henceforth abbreviated as Littmann 3200) electronic stethoscope (benchmark) next to the sensor, using a tight strap around the person’s torso. The recorded signals contained tidal breathing, deep breathing, one fit of two coughs, and speech segments. All these instants were manually annotated to perform a comparative analysis.

To compare the signals obtained with the sensor and the benchmark electronic stethoscope, the magnitude spectra of each signal acquired for the different auscultation points were determined. Moreover, four relevant audio descriptors were extracted to perform a descriptive and comparative analysis, namely spectral rolloff, brightness, centroid, and spread.

- Spectral rolloff estimates the amount of high frequency in the signal by finding the frequency below which a defined percentage of the total spectral energy is contained (in this case, 85%).
- Spectral brightness estimates the amount of high frequency in the signal by measuring the amount of energy above a cut-off frequency (in this case, 500 Hz, the frequency below which Littmann 3200 enhances the signal).
- Spectral centroid is the center of mass of the spectrum.
- Spectral spread indicates the spread of the spectrum around its mean value.

The spectral features were extracted using Matlab’s MIR Toolbox [24].

**Figure 6:**
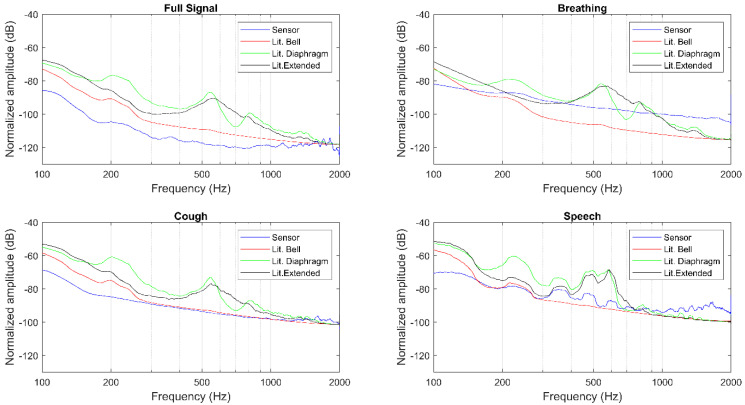
Magnitude spectra for position 18.

**Figure 7:**
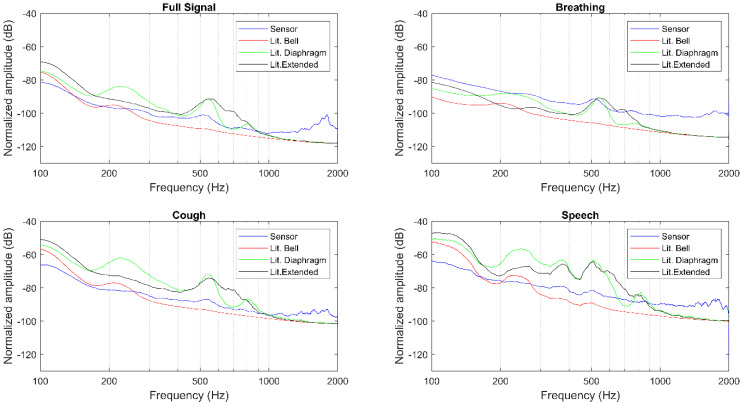
Magnitude spectra for position 7.

The magnitude spectra, as well as the described features, were extracted for the full signals, breathing, cough, and speech segments separately, as presented in Table 1, Table 2, Figure 6, and Figure 7. The Littmann 3200 (abbreviated as Lit. in Table 1 and Table 2) recordings were output using three different modes: in bell mode which amplifies sounds from 20 to 1000 Hz, and further emphasizes lower frequency sounds between 20 and 200 Hz; in diaphragm mode which amplifies sounds from 20 to 2000 Hz, and further emphasizes the sounds between 100 and 500 Hz; in extended range mode which amplifies sounds from 20 to 2000 Hz similar to the diaphragm mode, but provides more low frequency response between 50 and 500 Hz.

A qualitative evaluation of the magnitude spectra shows for both auscultation points and different segments considered that the sensor’s amplitude decays rapidly, but remains almost constant from 400 Hz to 2000 Hz (see Figure 6 and Figure 7). This behavior closely resembles the Littmann 3200 bell filter, while the diaphragm and extended filters present a less pronounced initial decay across all recordings. Moreover, the amplitude broadband of the sensor is generally higher than what is observed with the Littmann 3200′s different filtering modes.

## 4. Discussion and Conclusions

This work reports the design and characterization of a wearable stethoscope module which is intended to be integrated in a vest for multi-channel lung sound acquisition. The sensing unit is made of a contact microphone based on a piezoelectric sensor. The physical interface materials as well as the piezoelectric film itself are optimized to minimize the acoustic impedance mismatch at different junctions.

Based on a mechanical amplification approach that was introduced in [20], we have developed and fabricated sensor modules which are thin enough to be integrated in a garment. A novel assembly procedure to provide electrical contacts with both sides of the PVDF film has been introduced and successfully implemented. Transducer electronics have been designed by considering the challenges associated with high-impedance sensors and their shielding.

Assembled modules have been benchmarked with a commercial electronic stethoscope which is widely used in clinical settings. Spectral analysis of the signals acquired under different conditions such as breathing, coughing shows that the information content can be positioned between two different modes of the commercial stethoscope. Considering that this stethoscope has built-in digital filters, mainly to provide a hearing experience similar to that of the acoustic stethoscopes, we expect that the manifested differences between our device (which does not have any filters within its flat band) and the benchmarking device can substantially be minimized by the introduction of digital filters. For instance, in the case of speech for both positions it is observed that the form of the magnitude spectra acquired by the presented sensor and the benchmark stethoscope (with its highest bandwidth setting on, i.e., extended mode) are significantly similar except that for certain frequency windows the signal acquired with the benchmark stethoscope are further highlighted or suppressed. The frequency windows where the signals are amplified or attenuated are in agreement with the literature [25] which investigates the frequency response of the benchmark stethoscope. The work [25] more precisely indicates that at 550 Hz a low-quality resonance inducing an amplification is followed by an anti-resonance dip and at ~800 Hz a second peak is observed. Additionally, a low-pass filter (~18 dB/octave) is observed around 1 kHz. Applying these additional filters brings the spectra of the signals acquired by our device to an equivalent level to those by the benchmark stethoscope. Although the ultimate goal of the presented device is to process acquired sound signals and consequently provide a diagnostic aid to the clinicians, this additional feature provides a similar hearing experience to that of the benchmark stethoscope if the clinicians want to listen these sounds for comparison purposes.

The extracted features (Table 1 and Table 2), do not show a clear pattern for the two auscultation points based on either the full signal or the segmented events. In general, the sensor’s roll-off frequency is quite low and closer to the bell filter, apart from the breathing events recorded in auscultation point 7. This might indicate that the sensor is capturing more energy coming from lower frequencies. Another trend found in all recordings is the larger spectral spread of the sensor. The fact that the sensor presents a larger spectral spread might indicate that it is picking up more noise when compared to the electronic stethoscope. This is also verified with the magnitude spectra, with the higher amplitude broadband. As for the spectral brightness and centroid, for both auscultation points and the multiple segments considered, it can be observed that the diaphragm and extended filters present a very similar behavior, while the sensor is usually closer to the bell filter.

In general, the sensor characteristics are in between the Littmann 3200′s bell and diaphragm filters, but closer to the bell one. This means that the sensor emphasizes lower frequency sounds, as reported in the analysis of the extracted features. Moreover, the wideband noise of the sensor is relatively high, which demonstrates the necessity to develop and implement of relevant digital filters.

Future work will focus on applying high-pass filters on hardware level to suppress low frequencies, which may saturate the recording due to their high energy or may cause a reduction in the signal headroom; thus, compressing the response at higher frequencies [26]. Except for low frequencies below the frequency range of interest (100–2000 Hz), the sensor is quite linear; thus, we expect it will be able to capture abnormal and adventitious sounds without distortion. The sensitivity of the sensor can be further increased to improve signal-to-noise ratio, particularly where electronics noise is dominant; however, it reduces the linear range of operation. Such a trade-off can be considered as an option for use cases only cardiac sounds are of interest.

## Figures and Tables

**Table 1:** Extracted features with respiratory sounds recorded in position 7.

	Sensor	Rolloff (Hz)	Brightness (%)	Centroid (Hz)	Spread (Hz)
Full signal	Sensor	27.01	4.37	71.30	277.89
	Lit. Bell	52.25	1.90	42.56	169.71
	Lit. Diaphragm	96.37	3.73	64.90	193.57
	Lit. Extended	100.59	4.22	67.81	189.89
Breathing	Sensor	63.78	6.00	94.15	306.92
	Lit. Bell	20.02	3.12	48.08	215.43
	Lit. Diaphragm	40.83	4.63	63.21	224.87
	Lit. Extended	51.57	5.51	66.92	229.69
Cough	Sensor	14.16	2.75	45.52	210.94
	Lit. Bell	61.52	1.62	44.34	160.52
	Lit. Diaphragm	112.79	3.69	74.12	184.41
	Lit. Extended	103.52	3.94	70.49	181.34
Speech	Sensor	43.95	4.87	79.13	278.23
	Lit. Bell	114.75	1.61	55.28	159.72
	Lit. Diaphragm	243.65	4.51	112.80	194.11
	Lit. Extended	132.81	4.75	105.38	188.34

**Table 2:** Extracted features with respiratory sounds recorded in position 18.

	Sensor	Rolloff (Hz)	Brightness (%)	Centroid (Hz)	Spread (Hz)
Full signal	Sensor	15.63	2.49	41.28	198.10
	Lit. Bell	52.98	1.44	40.71	148.51
	Lit. Diaphragm	117.43	4.03	75.80	189.13
	Lit. Extended	101.87	4.03	69.09	183.11
Breathing	Sensor	22.40	4.25	65.83	250.77
	Lit. Bell	56.40	1.14	39.82	134.71
	Lit. Diaphragm	97.72	5.76	84.79	210.69
	Lit. Extended	91.80	6.17	83.34	203.04
Cough	Sensor	14.65	2.34	39.44	196.62
	Lit. Bell	49.80	1.84	44.56	169.08
	Lit. Diaphragm	114.26	3.63	72.19	191.17
	Lit. Extended	100.10	3.66	66.02	189.45
Speech	Sensor	20.51	3.75	65.67	254.94
	Lit. Bell	68.85	1.55	50.13	165.18
	Lit. Diaphragm	124.51	3.69	87.66	181.75
	Lit. Extended	120.61	3.94	81.30	180.99
